# A real-life treatment cohort of pancreatic neuroendocrine tumors: High-grade increase in metastases confers poor survival

**DOI:** 10.3389/fendo.2022.941210

**Published:** 2022-08-10

**Authors:** Wu-Hu Zhang, He-Li Gao, Wen-Sheng Liu, Yi Qin, Zeng Ye, Xin Lou, Fei Wang, Yue Zhang, Xue-Min Chen, Jie Chen, Xian-Jun Yu, Qi-Feng Zhuo, Xiao-Wu Xu, Shun-Rong Ji

**Affiliations:** ^1^ Center for Neuroendocrine Tumors, Fudan University Shanghai Cancer Center, Shanghai, China; ^2^ Department of Pancreatic Surgery, Fudan University Shanghai Cancer Center, Shanghai, China; ^3^ Department of Oncology, Shanghai Medical College, Fudan University, Shanghai, China; ^4^ Shanghai Pancreatic Cancer Institute, Shanghai, China; ^5^ Pancreatic Cancer Institute, Fudan University, Shanghai, China; ^6^ The First People’s Hospital of Changzhou, The Third Affiliated Hospital of Soochow University, Changzhou, China

**Keywords:** pancreatic neuroendocrine tumors, Ki67 index variation, grade increase, preoperative neoadjuvant treatment, prognosis

## Abstract

**Background:**

Tumor grade determined by the Ki67 index is the best prognostic factor for pancreatic neuroendocrine tumors (PanNETs). However, we often observe that the grade of metastases differs from that of their primary tumors. This study aimed to investigate the frequency of grade changes between primary tumors and metastases, explore its association with clinical characteristics, and correlate the findings with the prognosis.

**Methods:**

Six hundred forty-eight patients with pancreatic neuroendocrine neoplasms treated at Fudan University Shanghai Cancer Center were screened for inclusion, and 103 patients with PanNETs who had paired primary tumors and metastases with an available Ki67 index were included. Re-evaluation of Ki67 was performed on 98 available samples from 69 patients.

**Results:**

Fifty cases (48.5%) had a Ki67 index variation, and 18 cases (17.5%) displayed a grade increase. Metachronous metastases showed significantly higher Ki67 index variation than synchronous metastases (*P*=0.028). Kaplan–Meier analyses showed that high-grade metastases compared to low-grade primary tumors were significantly associated with decreased progression-free survival (PFS, *P*=0.012) and overall survival (OS, *P*=0.027). Multivariable Cox regression analyses demonstrated that a low-grade increase to high-grade was an unfavorable and independent prognostic factor for PFS and OS (*P*=0.010, and *P*=0.041, respectively).

**Conclusions:**

A high-grade increase in metastases was an unfavorable predictor of PanNETs, which emphasized the importance of accurate pathological grading and could provide a reference for clinical decision-making.

## Introduction

Pancreatic neuroendocrine tumors (PanNETs) are a rare and heterogeneous group of tumors arising from the neuroendocrine system, and their prevalence has markedly increased over the last four decades (1–3). The clinical courses of PanNETs are highly variable, and their prognosis differs widely, ranging from indolent tumors with reasonable survival to distant metastases of an aggressive nature with a poor prognosis. Up to 80% of patients with PanNETs present with metastases at the time of diagnosis, mainly to the liver (4–6), whose median survival is only 23 months, compared to 124 months for localized disease (7). PanNETs with metastases require a multidisciplinary treatment approach, including surgery (8), somatostatin analogs (SSAs), targeted therapy, namely, everolimus and sunitinib (9, 10), chemotherapy (11), and/or peptide receptor radionuclide therapy (12). Surgery remains the only curative treatment for these patients, and patients can benefit from neoadjuvant treatment (NAT).

For better prognostic stratification, the European Neuroendocrine Tumor Society (ENETS) originally proposed a three-tiered grading system for gastroenteropancreatic neuroendocrine neoplasms (GEP-NENs) in 2006, and the World Health Organization (WHO) adopted the classification in 2010, which was mainly based on the Ki67 index and mitotic index (13–15). The WHO modified the classification in 2017 and divided pancreatic neuroendocrine neoplasms (PanNENs) into well-differentiated neuroendocrine tumors (NETs) and poorly differentiated neuroendocrine carcinoma (NEC). Given that the Ki67 index has been proven to be the most reliable and best prognostic factor of PanNENs (16), the 2017 WHO classification requires its use and strongly recommends careful evaluation of the Ki67 index. Accordingly, well-differentiated NETs are further divided into the following grades by the Ki67 index: G1: <3%; G2: 3–20%; G3: >20% (17, 18).

Grade currently plays a pivotal role in the clinical management of patients with NETs, especially PanNETs (19). However, concerns exist regarding Ki67 index variation and grade differences between the primary tumors and metastases (20–22). We also observed that the grade of metastases differed from that of their primary tumors in PanNET patients in clinical setting. Here, we set out to investigate the frequency of grade changes between primary tumors and metastases in PanNETs, to explore the association between clinical characteristics and grade changes and to determine whether grade changes correlate with patient outcomes. A better understanding of grade changes could add to our understanding of the heterogeneity between primary tumors and metastases in PanNETs and contribute to clinical decision-making.

## Methods

### Patient population

A total of 648 patients with pathology-confirmed PanNENs who were treated at Fudan University Shanghai Cancer Center (FUSCC) between January 1, 2006, and February 1, 2020, were screened for inclusion. Exclusion criteria for the study cohort were familial syndromes, no metastatic disease, and no pathology reports available or consultation with our institution. Then, 169 patients with metastatic PanNETs were included for further eligibility evaluation. Next, 103 patients who had paired primary tumors and metastases with an available Ki67 index were identified as the study cohort. To assess the selection bias due to the inclusion of patients who had paired primary tumors and metastases with an available Ki67 index, the remaining 66 patients who did not have paired tumors with an available Ki67 index were identified as the bias-control group. Additionally, to explore whether NETs evolved to NEC, 15 patients with metastatic PanNEC were served as additional data.

The study cohort consisted of 91 patients who underwent resection and 12 patients who underwent needle biopsy or laparotomy with biopsy. In the resection cohort, 74 and 17 patients had synchronous and metachronous metastases, respectively. The biopsy cohort consisted of 12 patients with synchronous metastases. Metastatic PanNETs patients with high-risk factors, including relatively large tumors, blood vessels and adjacent organ invasion were routinely received preoperative NAT. And surgical resection was considered after multidisciplinary discussion if the tumors stabilized or regressed. Pathology reports, including Ki67 index, size of the tumor, perineural, lymphatic, and microvascular invasion, and clinical data, including sex, age, operative procedures, treatment information, and follow-up information, were retrospectively reviewed from the medical record database.

Patients were observed at 3- to 6-month intervals following resection or biopsy and underwent physical examination, laboratory investigation and contrast-enhanced computed tomography or magnetic resonance imaging of the chest/abdomen/pelvis. PFS was defined as the time from the date of resection or biopsy to the date of progression. OS was defined as the time from the date of resection or biopsy to the date of either death (event) or the last follow-up (censored).

Tumors were restaged and regraded based on the 8th edition American Joint Committee on Cancer (AJCC 8^th^) tumor-node-metastasis (TNM) staging system and 2019 WHO classification. Metastatic diseases were divided into locoregional (nodal or mesenteric involvement) and distant metastases (liver, peritoneum or other sites). Liver metastases (LM) were classified into type I, II and III (23). Type I LM were confined to one liver lobe or two adjacent segments that could be removed by a standard anatomical resection. Type II LM primarily affected one lobe, with smaller satellites contralaterally, and could be managed surgically, including ablative approaches. Type III LM were diffuse, multifocal liver metastases that could not be treated surgically.

This retrospective study was approved by the institutional research ethics committee of FUSCC, and informed consent was obtained from the patients included in this study.

### Immunohistochemistry and re-evaluation

Formalin-fixed, paraffin-embedded (FFPE) samples of 69 patients were available, including 63 primary tumors, 5 involved lymph nodes, and 30 LM. Slides of these samples were subjected to Ki67 immunohistochemical staining (MIB-1, Dako Corporation, CA, USA) using a two-step method as previously described (24–27). Detailed experimental procedures were described in the **Supplementary Materials** [available in a digital data repository (28)]. Additionally, TP53 mutation was associated with Ki67 index variation and might be a possible biologic mechanism for high-grade transition (22, 29, 30). Therefore, we selected FFPE samples of 27 patients for p53 immunohistochemical staining (ab1101, Abcam, Cambridge, UK). Abnormal p53 expression was defined as complete absence or more than 10% of tumor cells presented with moderate to strong nuclear positivity (30–32).

### Statistical analyses

Statistical analyses were performed using SPSS (version 25.0, IBM, Armonk, NY, USA). Continuous variables were compared by nonparametric tests or Student’s t-test. Categorical variables were compared by Pearson’s chi-squared test or Fisher’s test. The Kaplan–Meier method and log-rank test were used to estimate significant differences in survival. Cox regression analyses were performed to identify independent prognostic factors of PFS and OS. Variables significantly associated with PFS or OS in the univariable analysis were included in the multivariable analysis. Logistic regression models were used to control confounding variables. Two-sided *P values* of <0.05 were considered statistically significant. Figures were drawn with GraphPad Prism (version 8.0, San Diego, CA, USA, www.graphpad.com).

## Results

### Patient characteristics

**Figure 1:**
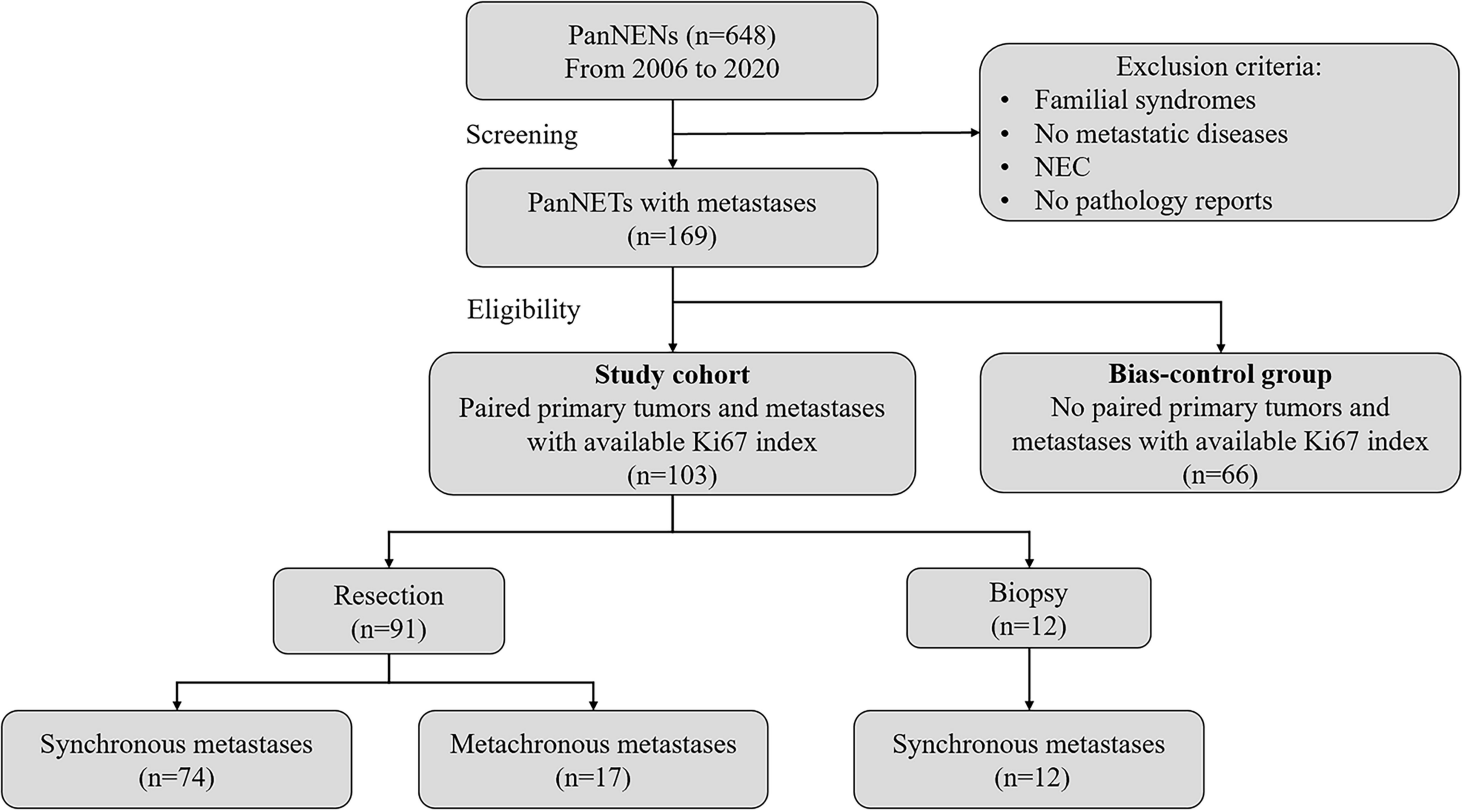
Flow diagram of the study cohort. Out of the 648 patients with PanNENs, 169 patients with metastatic PanNETs were included for further eligibility evaluation. Then 103 patients had paired primary tumors and metastases with an available Ki67 index were identified as the study cohort, and the remaining 66 patients were identified as the bias-control group. The study cohort consisted of 91 patients underwent resection and 12 patients experienced biopsy. In resection cohort, 74 and 17 patients had synchronous and metachronous metastases, respectively. And biopsy cohort consisted of 12 patients with synchronous metastases.

A total of 103 patients who had paired primary tumors and metastases with an available Ki67 index were identified as the study cohort. These patients were divided into a resection cohort and a biopsy cohort (**Figure 1**). The clinicopathological characteristics and treatment information of the study cohort, resection cohort, and biopsy cohort were analyzed and are summarized in **Table 1**. In the study cohort, 4 patients had functional tumors, and their specific information is shown in **Supplementary Table S1**. The grade at first diagnosis was distributed as follows: G1 NET-10 (9.7%), G2 NET-76 (73.8%), and G3 NET-17 (16.5%). A total of 20 patients underwent pancreatoduodenectomy with or without LM resection, 66 patients underwent distal pancreatectomy with or without LM resection, 4 patients underwent total pancreatectomy with or without LM resection, and one patient underwent middle pancreatectomy with LM resection. Furthermore, 3 patients underwent laparotomy with biopsy, and 9 patients underwent needle biopsy. For sites of metastases, 92 patients (89.3%) had LM, 9 patients (8.7%) had nodal or mesenteric metastases, and 2 patients (1.9%) had peritoneum or other distant metastases. In addition, 86 patients (83.5%) were diagnosed with synchronous metastases, and 17 patients (16.5%) developed metachronous metastases.

**Table:** Table 1. Demographics and clinical characteristics of the study cohort, resection and biopsy cohort.

Characteristics	Study cohort (n=103)	Resection cohort (n=91)	Biopsy cohort(n=12)
	No. (%)	No. (%)	No. (%)
**Gender**			
Male	50 (48.5)	42 (46.2)	8 (66.7)
Female	53 (51.5)	49 (53.8)	4 (33.3)
**Age, years, median**	51	50.5	49.5
**Tumor size, mm**			
Mean (SD)	48.7 (3.0)	47.7 (3.0)	56.8 (3.4)
Median (range)	40.0 (8.0-160.0)	40.0 (8.0-160.0)	47.0 (20.0-120.0)
**Location**			
Head	28 (27.2)	22 (24.2)	6 (50.0)
Neck	6 (5.8)	5 (5.5)	1 (8.3)
Body	15 (14.6)	14 (15.4)	1 (8.3)
Tail	25 (24.3)	23 (25.3)	2 (16.7)
Body-Tail	29 (28.2)	27 (29.7)	2 (16.7)
**Functional**			
Yes	4 (3.9)	4 (4.4)	0 (0.0)
No	99 (96.1)	87 (95.6)	12 (100.0)
**Lymph node positive**	n=88	n=88	NA
Yes	48 (54.5)	48 (54.5)	
No	40 (45.5)	40 (45.5)	
**Perineural invasion**	n=84	n=84	NA
Yes	41 (48.8)	41 (48.8)	
No	43 (51.2)	43 (51.2)	
**Microvascular invasion**	n=84	n=84	NA
Yes	57 (67.9)	57 (67.9)	
No	27 (32.1)	27 (32.1)	
**CgA^a^**	n=102	n=90	n=12
Positive	97 (95.1)	86 (95.6)	11 (91.7)
Negative	5 (4.9)	4 (4.4)	1 (8.3)
**Syn^a^**	n=101	n=89	n=12
Positive	100 (99.0)	88 (98.9)	12 (100.0)
Negative	1 (1.0)	1 (1.1)	0 (0.0)
**DAXX^a^**	n=38	n=38	NA
Positive	34 (89.5)	34 (89.5)	
Negative	4 (10.5)	4 (10.5)	
**ATRX^a^**	n=36	n=36	NA
Positive	32 (88.9)	32 (88.9)	
Negative	4 (11.1)	4 (11.1)	
**SSTR^a^**	n=57	n=55	n=2
Positive	53 (93.0)	51 (92.7)	2 (100.0)
SSTR2	38 (66.7)	36 (65.4)	2 (100.0)
SSTR2+SSTR5	15 (26.3)	15 (27.3)	0 (0.0)
Negative	4 (7.0)	4 (7.3)	0 (0.0)
**NSE classification**	n=84	n=76	n=8
High	26 (31.0)	22 (28.9)	4 (50.0)
Low	58 (69.0)	54 (71.1)	4 (50.0)
**PROGRP classification**	n=46	n=43	n=3
High	8 (17.4)	7 (16.3)	1 (33.3)
Low	38 (82.6)	36 (83.7)	2 (66.7)
**Metastases**			
Site			
Liver	92 (89.3)	81 (89.0)	11 (91.7)
Nodal/mesenteric	9 (8.7)	9 (9.9)	0 (0.0)
Peritoneum/others	2 (1.9)	1 (1.1)	1 (8.3)
Type			
Synchronous	86 (83.5)	74 (81.3)	12 (100.0)
Metachronous	17 (16.5)	17 (18.7)	0 (0.0)
**AJCC 8^th^ TNM stage**			
I	1 (1.0)	1 (1.1)	0 (0.0)
II	12 (11.7)	12 (13.2)	0 (0.0)
III	10 (9.7)	10 (11.0)	0 (0.0)
IV	80 (77.7)	68 (74.7)	12 (100.0)
**WHO classification^b^**			
G1	10 (9.7)	9 (9.9)	1 (8.3)
G2	76 (73.8)	68 (74.7)	8 (66.7)
G3	17 (16.5)	14 (15.4)	3 (25.0)
**Operating methods**			
Pancreatoduodenectomy Distal Pancreatectomy Total pancreatectomy Total pancreatectomy with LM resection Middle pancreatectomy with LM resection Pancreatoduodenectomy with LM resection Distal Pancreatectomy with LM resection Laparotomy with biopsy. Needle biopsy	6 (5.8) 15 (14.6) 2 (1.9) 2 (1.9) 1 (1.0) 14 (13.6) 51 (49.5) 3 (2.9) 9 (8.7)	6 (6.6) 15 (16.5) 2 (2.2) 2 (2.2) 1 (1.1) 14 (15.4) 51 (56.0) 0 (0.0) 0 (0.0)	0 (0.0) 0 (0.0) 0 (0.0) 0 (0.0) 0 (0.0) 0 (0.0) 0 (0.0) 3 (25.0) 9 (75.0)
**Treatment**			
Neoadjuvant treatment SSAs with or without targeted therapy CAPTEM with or without targeted therapy SSAs+CAPTEM with or without targeted therapy Locoregional treatment Other chemotherapy Targeted therapy Others	n=33 9 (27.3) 11 (33.3) 7 (21.2) 1 (3.0) 3 (9.1) 1 (2.0) 1 (3.0)	n=33 9 (27.3) 11 (33.3) 7 (21.2) 1 (3.0) 3 (9.1) 1 (3.0) 1 (2.0)	NA
Adjuvant treatment SSAs with or without targeted therapy CAPTEM with or without targeted therapy SSAs+CAPTEM with or without targeted therapy Locoregional treatment Other chemotherapy Targeted therapy Others	n=78 35 (44.9) 6 (7.7) 6 (7.7) 12 (15.4) 4 (5.1) 15 (19.2) 0 (0.0)	n=78 35 (44.9) 6 (7.7) 6 (7.7) 12 (15.4) 4 (5.1) 15 (19.2) 0 (0.0)	NA
Treatment for patients with biopsy SSAs with or without targeted therapy CAPTEM with or without targeted therapy SSAs+CAPTEM with or without targeted therapy Locoregional treatment Other chemotherapy Targeted therapy Others	n=12 4 (33.3) 2 (16.7) 3 (25.0) 1 (8.3) 0 (0.0) 2 (16.7) 0 (0.0)	NA	n=12 4 (33.3) 2 (16.7) 3 (25.0) 1 (8.3) 0 (0.0) 2 (16.7) 0 (0.0)

CgA, chromogranin; Syn, synaptophysin; DAXX, death domain associated protein; ATRX, alpha-thalassemia/mental retardation X-linked; SSTR, somatostatin receptor; NSE, neuron specific enolase; PROGRP, progastrin releasing peptide; AJCC, American Joint Committee on Cancer; WHO, World Health Organization; ^**a**^The expression in primary tumors; **^b^** Grade at first diagnosis; LM, liver metastases; SSAs, somatostatin analogs; CAPTEM, capecitabine and temozolomide; NA, not available.

### Ki67 index variation and grade changes between primary tumors and metastases

**Figure 2:**
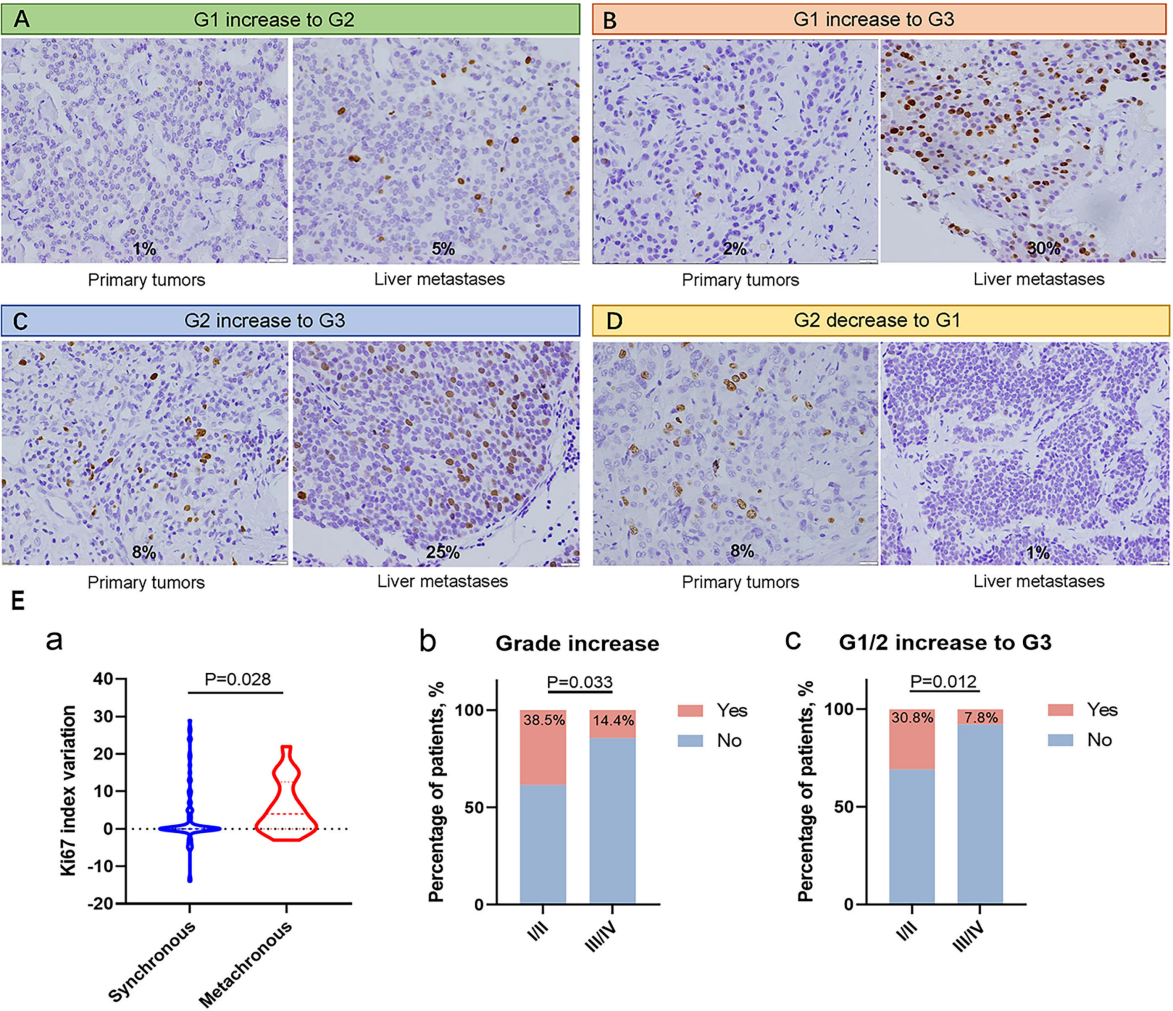
**(A–D)** Immunohistochemistry staining of Ki67 in available paired primary tumors and metastases, showing G1 increase to G2, G1 increase to G3, G2 increase to G3, and G2 decrease to G1, respectively. Magnification: 400×. **(E)** Association among clinicopathological characteristics, Ki67 index variation and grade changes. (a) Metachronous metastases showed higher Ki67 index variation than synchronous metastases. (b, c) Patients with AJCC 8^th^ stage I/II showed higher frequency of grade increase, and G1/G2 increase to G3 than patients with AJCC 8^th^ stage III/IV.

In the study cohort, 50 cases (48.5%) showed variance in the Ki67 index between primary tumors and metastases, and the median variation in the Ki67 index in metastases compared to primary tumors was 0%, ranging from -14% to +29% (**Table 2**). The Ki67 variation led to grade changes between the primary tumors and metastases; 18 patients (17.5%) had a grade increase, and a grade decrease was observed in 6 patients (5.8%). Among those with grade increases, 7 patients (6.8%) changed from G1 to G2, 4 patients (3.9%) changed from G1 to G3, and 7 patients (6.8%) changed from G2 to G3 (**Figures 2A–D**). The characteristics of the eleven patients with G1 or G2 (G1/G2) increase to G3 are shown in **Supplementary Table S2**. In addition, grade changes between the primary tumors and metastases were found in 21 cases (23.1%) in the resection cohort and 3 cases (25.0%) in the biopsy cohort.

**Table:** Table 2. Ki67 index variation and grade changes in metastases compared to primary tumors in PanNETs.

Ki67 index variation	Study cohort (n=103)	Resection cohort (n=91)	Biopsy cohort (n=12)
Patient number (%)	50 (48.5)	43 (47.3)	7 (58.3)
Delta Ki67 index, median (range)	0 (-14 to +29)	0 (-14 to +29)	0.5 (-5 to +27)
**Tumor grade**			
Stable, n (%)	79 (76.7)	70 (76.9)	9 (75.0)
Grade increase, n (%)	18 (17.5)	15 (16.5)	3 (25.0)
G1 increase to G2	7 (6.8)	6 (6.6)	1 (8.3)
G1 increase to G3	4 (3.9)	4 (4.4)	0 (0)
G2 increase to G3	7 (6.8)	5 (5.5)	2 (16.7)
Grade decrease, n (%)			
G2 decrease to G1	6 (5.8)	6 (6.6)	0 (0)

### Association among clinical characteristics, Ki67 index variation, and grade changes

Metachronous metastases showed significantly higher Ki67 index variation than synchronous metastases (P=0.028, **Figures 2Ea**). Meanwhile, metachronous metastases had a higher proportion of G1/G2 increase to G3 than synchronous metastases, although the difference was not significant (P=0.060). Patients with AJCC 8^th^ stage I/II showed a higher frequency of grade increase and G1/G2 increase to G3 than patients with AJCC 8^th^ stage III/IV (P=0.033 and P=0.012, respectively, **Figures 2Eb, c**), which was consistent with the result that metachronous metastases showed higher Ki67 index variation. In the eleven patients with a G1/G2 increase to G3, all 4 patients with metachronous metastases and AJCC 8^th^ stage II at first diagnosis showed a G2 increase to G3; among the 7 patients with synchronous metastases and AJCC 8^th^ stage IV at first diagnosis, 4 patients had a G1 increase to G3, and the remaining 3 patients had a G2 increase to G3, indicating that patients with stage IV were more likely to have a more severe grade increase (**Supplementary Table S2**).

### Ki67 index variation and grade changes in advanced patients who received NAT

Increasing evidence supports the application of NAT in advanced PanNETs (33, 34). Among the 68 advanced patients (AJCC 8^th^ stage IV) who underwent surgical resection, 31 patients received NAT, and 37 cases did not receive NAT. The clinicopathological characteristics and treatment information of these patients are summarized in **Supplementary Table S3**. Of the 31 patients receiving NAT, 9 patients (29.0%) received SSAs with or without targeted therapy, 11 patients (35.5%) received capecitabine and temozolomide (CAPTEM) with or without targeted therapy, 7 patients (22.6%) received SSAs+CAPTEM with or without targeted therapy, a case (3.2%) received locoregional treatment, 2 patients (6.5%) received other chemotherapeutic regimens, and one patient (3.2%) received targeted therapy. All patients treated with NAT had LM, including 2 patients (6.5%) with type I LM, 2 patients (6.5%) with type II LM, and 27 patients (87.1%) with type III LM. Patients who received NAT presented a significantly higher proportion of type III LM than those who did not receive NAT (87.1% vs. 50.0%, P=0.009).

These patients achieved the effect of tumor stabilization or regression after receiving NAT and could be considered for surgery after multidisciplinary discussion. Among them, 8 patients underwent pancreaticoduodenal resection with LM, and 23 cases underwent distal pancreaticoduodenal resection with LM. Additionally, advanced patients who received NAT presented a significantly higher proportion of grade increase (25.8% vs. 5.4%, P=0.018) than those who did not receive NAT (**Table S3**).

### Survival analyses

**Figure 3:**
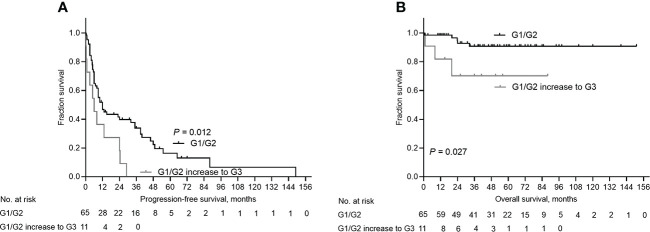
Kaplan-Meier curves depicting progression-free survival (PFS) and overall survival (OS) for patients G1/G2 increase to G3. **(A)** Patients with G1/G2 increase to G3 had decreased PFS than patients with stable G1/2 (*P*=0.012). **(B)** Patients with G1/G2 increase to G3 had decreased OS than patients with stable G1/2 (*P*=0.027).

Next, we performed survival analyses to determine whether grade changes affect the patient prognosis. In the study cohort, the median follow-up time was 48.0 months [95% confidence interval (CI): 39.70-56.30], and the median PFS was 10.0 months (95% CI: 6.33-13.67). The Kaplan–Meier survival probability estimates of 1-year, 3-year, and 5-year progression-free survival were 41.7%, 18.4%, and 6.8%, respectively. The Kaplan–Meier survival probability estimates of 1-year, 3-year, and 5-year overall survival were 85.4%, 52.4%, and 28.2%, respectively (**Figure S1**). Of importance, G1/G2 increase to G3 was significantly associated with shorter PFS and OS than stable G1/G2 (P=0.012 and P=0.027, respectively, **Figure 3**). G2 increase to G3 was associated with shorter PFS and OS, although it was not statistically significant (P=0.115, and P=0.064, respectively, **Supplementary Figures S2A, B**). In addition, G2 decrease to G1 was associated with a longer OS, although the difference was not statistically significant (P=0.445, **Supplementary Figure S2D**).

Univariable analyses demonstrated that biopsy, NAT, and a low-grade increase to high-grade were associated with a shorter PFS (HR=3.443, 95% CI: 1.809–6.552, P=0.000; HR=2.186, 95% CI: 1.284-3.720, P=0.004, and HR=2.281, 95% CI: 1.162-4.479, P=0.017, respectively, **Table 3**). Biopsy and a low-grade increase to high-grade were associated with shorter OS (HR=6.862, 95% CI: 1.969-23.916, P=0.002; and HR=4.418, 95% CI: 1.051-18.578, P=0.043, respectively). Multivariable survival analyses indicated that NAT and a low-grade increase to high-grade were independent prognostic factors for PFS (HR=2.756, 95% CI: 1.474-5.153, P=0.001; and HR=2.695, 95% CI: 1.273-5.706, P=0.010, respectively). In addition, a low-grade increase to high-grade was an independent prognostic factor for OS (HR= 4.565, 95% CI: 1.063-19.612, P=0.041).

**Table:** Table 3. Univariable and multivariable analysis of factors for progression-free survival and overall survival in the study cohort.

Factors	Progression-free Survival						Overall Survival					
	Univariable			Multivariable			Univariable			Multivariable		
	HR (95% CI)	*P-*value		HR (95% CI)	*P-*value		HR (95% CI)	*P*-value		HR (95% CI)	*P*-value	
Gender: male *vs* female	0.787 (0.505-1.227)		0.291				1.844 (0.563-6.040)		0.312			
Age: <51 *vs* ≥51 years	0.745 (0.480-1.156)		0.189				0.474 (0.146-1.543)		0.215			
Tumor size: <40 *vs* ≥ 40mm	1.135 (0.720-1.790)		0.584				0.785 (0.262-2.350)		0.665			
Tumor location: head *vs* neck, body and tail	1.582 (0.967-2.590)		0.068				1.058 (0.287-3.892)		0.933			
Functional: no *vs* yes	0.408 (0.126-1.321)		0.135				1.443 (0.185-11.283)		0.726			
Lymph node positive: no *vs* yes	1.128 (0.692-1.838)		0.628				1.892 (0.472-7.592)		0.368			
Perineural invasion: no *vs* yes	1.103 (0.668-1.823)		0.701				1.599 (0.376-6.798)		0.525			
Microvascular invasion: no *vs* yes	0.738 (0.431-1.263)		0.268				3.938 (0.483-32.072)		0.200			
CgA: negative *vs* positive	0.511 (0.206-1.271)		0.149				0.659 (0.084-5.158)		0.691			
Syn: negative *vs* positive	0.351 (0.048-2.575)		0.303				0.167 (0.021-1.320)		0.090			
DAXX: negative *vs* positive	1.411 (0.332-6.007)		0.641				23.321 (0-3.78E+10)		0.771			
ATRX: negative *vs* positive	2.470 (0.705-8.652)		0.157				31.539 (0-1.10E+7)		0.596			
SSTR: negative *vs* positive	0.512 (0.180-1.456)		0.210				22.359 (0-1.26E+10)		0.762			
Metastases site: liver *vs* nodal/mesenteric *vs* peritoneum/others	0.821 (0.479-1.409)		0.475				0.608 (0.096-3.864)		0.598			
Metastases type: synchronous *vs* metachronous	0.816 (0.462-1.443)		0.485				0.270 (0.034-2.125)		0.214			
Surgery: yes *vs* biopsy	3.443 (1.809-6.552)		**0.000**	NA		NA	6.862 (1.969-23.916)		**0.002**	4.254 (0.804-22.507)		0.088
Neoadjuvant treatment: no *vs* yes	2.186 (1.284-3.720)		**0.004**	2.756 (1.474-5.153)		**0.001**	0.743 (0.151-3.669)		0.716			
Grade changes: G1/G2 *vs* G1/G2 increase to G3	2.281 (1.162-4.479)		**0.017**	2.695 (1.273-5.706)		**0.010**	4.418 (1.051-18.578)		**0.043**	4.565 (1.063-19.612)		**0.041**

HR, hazard ratio; CI, confidence interval; CgA, chromogranin; Syn, synaptophysin; DAXX, death domain associated protein; ATRX, alpha-thalassemia/mental retardation X-linked; SSTR, somatostatin receptor; NSE, neuron specific enolase; PROGRP, progastrin releasing peptide; TNM, tumor–node–metastasis; NA, not available; bold values indicate statistical significance.

### Association between p53 expression and Ki67 index variation

To further explore the association between high-grade transition and TP53 mutation, we performed p53 immunohistochemistry staining. Four out of 27 patients (14.8%) showed different p53 expression pattern between primary tumors and liver metastases, which was correlated with higher Ki67 index variation (*P*=0.031, **Supplementary Figure S5**)

## Discussion

In this retrospective study, 648 patients with PanNENs were screened, and 103 patients with advanced PanNETs who had paired primary tumors and metastases were identified as the study cohort. We described the Ki67 index variation and tumor grade changes in PanNETs, explored the association between the clinical characteristics and grade changes, and determined whether the grade changes predicted the clinical prognosis. The findings of this study could help improve our understanding of the heterogeneity of PanNETs and contribute to clinical decision-making.

In this study, 48.5% of cases had Ki67 index variation, 23.3% displayed grade changes, and 17.5% showed a grade increase. Of note, 10.7% of cases had high-grade metastases compared to low-grade primary tumors. For the associations among clinical characteristics, Ki67 index variation, and grade changes, metachronous metastases presented with higher Ki67 index variation than synchronous metastases. Patients with early-stage disease showed a higher frequency of grade increase and G1/G2 increase to G3 than patients with advanced-stage disease, indicating that early-stage tumors had more time to evolve, which was consistent with previous observations that NETs dedifferentiated over time. Additionally, G1 increase to G3 only presented in patients with stage IV and synchronous metastases, indicating that advanced tumors had a worse evolution than less advanced tumors. In the study cohort, all patients had only one primary tumor except one patient with two primary tumors, and the Ki67 values of the two primary tumors were both 5%. Therefore, the Ki67 index heterogeneity among primary tumors was not analyzed.

Previous studies reported that 35.3%-63% of metastatic GEP-NENs showed higher Ki-67 index at LM than primary site (35–37), and 7.5%-39% presented a grade increase (20, 21, 38–40). As for longitudinal increase, about 58.6%-65.1% of NEN patients showed an increase in Ki67 index, and nearly 28% showed upgrade when progression (41–44). Shi et al. reported that nearly two-thirds of small intestinal NETs had grade increase in LM (45). As for PanNENs, several studies described that 62.5%-63.6% patients with PanNETs had an increased grade when the disease evolved over time (22, 46). Alexandraki et al. analyzed 264 PanNENs and showed that 15 patients (5.7%) developed an increase in Ki67 during disease course (31). However, this study also included patients with multiple endocrine neoplasia type 1, and did not provide information about the 264-patient cohort. Given the strong heterogeneity of NENs, the results obtained in relatively homogeneous tumors would be more powerful. Therefore, this work aimed to explore the heterogeneity between primary tumors and metastases in a homogeneous and large-scale patient cohort of PanNETs, mainly to characterize the grade changes and correlate the findings with patient prognosis, also providing real-life data from the clinical treatment.

The “transformed” NETs that would evolve to either NET G3 or NEC has aroused interest. Several studies investigated this phenomenon (47–51) and proposed that the boundary between NETs and NEC was blurred. In this study, we observed that NET G1/G2 could evolve to NET G3, but did not find the transition from NETs to NEC. The concept that well-differentiated NETs would develop to poor-differentiated NEC probably needed further investigation.

Additionally, a suspicion of a grade increase in metastatic sites compared to primary tumors might constitute a negative prognostic factor (52). Therefore, we performed survival analyses and found that high-grade metastases compared to low-grade primary tumors were significantly associated with decreased PFS and OS. Multivariable survival analyses indicated that a low-grade increase to high-grade was an independent prognostic factor for PFS and OS. Based on the above results, we concluded that PanNET patients with metastases, whether synchronous or metachronous, would have a grade increase in metastases compared to their primary tumors. Because of the longer clinical course of metachronous metastases, the probability of a grade increase was higher than among those with synchronous metastases. In addition, G1 increase to G3, a progression of two grades, but only in AJCC 8^th^ stage IV tumors, suggesting that advanced tumors had a worse evolution than less advanced tumors.

Furthermore, Cox regression analysis showed that patients who received NAT had a shorter PFS, whereas there was no significant difference in OS. It should be noted that patients with high-risk factors, were routinely received preoperative NAT. Surgical resection was considered after multidisciplinary discussion if the tumors stabilized or regressed. It was obvious that patients with high-risk factors had a worse prognosis, but the results did not show any significant difference in OS compared to those without high-risk factors, indicating the potential effectiveness of preoperative NAT in patients with metastatic PanNETs.

Our results revealed the poor survival of patients with a high-grade increase in metastases, indicating the necessity of close follow-up and early intervention. Those patients with high-grade increase warrant different therapeutic strategies. In addition, this study also supported the use of multiple point puncture and accurate pathological grading for PanNET patients in metastatic lesions or when disease progression.

The possible underlying mechanism of Ki67 index heterogeneity and high-grade transition might due to the polyclonal tumor origin of NENs, with different mutational events, microenvironmental context, and/or epigenetic divergence between and within tumors (45). During the progression course, the selection of subclonal populations with a higher proliferative index (20), and de-reprograming would happen. In this study, higher Ki67 index variation was correlated with changes in p53 expression pattern, implying the TP53 mutation was probably involved in the high-grade progression. Additionally, treatment effects and therapy resistance might also contribute to the transition towards higher grade (44). The above hypotheses warrant further research.

Several limitations existed in the current study. First, all retrospective studies have inherent limitations. Second, the prognosis of most patients with PanNETs is good (53), but the median follow-up time of this study was relatively short. The follow-up data were available up until February 1, 2022, by which time only 13 cases (12.6%) had died. Those who had not died were considered right-censored, which might underestimate the overall survival time. Third, this study had selection bias of low to intermediate degrees due to the nonsignificant difference in survival between the study cohort and the bias control group. In addition, we did not explore the molecular mechanism of the clinical phenomenon that low-grade primary tumors would increase to high-grade metastases. To address this limitation, we are conducting another study to detect paired low-grade primary tumors and high-grade metastases using whole-exome sequencing to identify the mutant genes driving the grade increase of metastases and to investigate its molecular mechanism.

Overall, this study found that 17.5% of patients with metastatic PanNETs had a grade increase in metastases compared to their primary tumors. A high-grade increase in metastases was an unfavorable prognostic factor for PFS and OS, which could provide a useful reference for clinical decision-making.

## Data availability statement

The original contributions presented in the study are included in the article/**Supplementary Material**. Further inquiries can be directed to the corresponding authors.

## Ethics statement

The studies involving human participants were reviewed and approved by institutional research ethics committee of Fudan University Shanghai Cancer Center. The patients/participants provided their written informed consent to participate in this study.

## Author contributions

W-HZ, JC, X-JY, X-WX and S-RJ conceived the original idea of the study. H-LG, W-SL, and YQ contributed to sample preparation and data collection. WH-Z, ZY, XL, and FW conducted all statistical analyses. WH-Z, Q-FZ, ZY, YZ, and X-MC contributed to the implementation of the research and interpretation of data. All authors discussed the results, helped prepare and shape the research and gave final approval of the manuscript to be published.

## Funding

This work was jointly supported by the Rare Tumor Research Special Project of the National Natural Science Foundation of China (82141104), National Natural Science Foundation of China (U21A20374), Shanghai Municipal Science and Technology Major Project (21JC1401500), Scientific Innovation Project of Shanghai Education Committee (2019-01-07-00-07-E00057), Clinical Research Plan of Shanghai Hospital Development Center (SHDC2020CR1006A), Xuhui District Artificial Intelligence Medical Hospital Cooperation Project (2021–011), Shanghai Municipal Science and Technology Commission (20ZR1471100), National Natural Science Foundation of China (No. 82141129, 82173281, 82173282, 82172577, 82172948, 81972725, 81972250, and 81871950), Commission of Health and Family Planning (2018YQ06), and Shanghai Municipal Science and Technology Commission (19QA1402100).

## Conflict of interest

The authors declare that the research was conducted in the absence of any commercial or financial relationships that could be construed as a potential conflict of interest.

## Publisher’s note

All claims expressed in this article are solely those of the authors and do not necessarily represent those of their affiliated organizations, or those of the publisher, the editors and the reviewers. Any product that may be evaluated in this article, or claim that may be made by its manufacturer, is not guaranteed or endorsed by the publisher.

## References

[1] Halfdanarson TR Rabe KG Rubin J Petersen GM. Pancreatic neuroendocrine tumors (Pnets): Incidence, prognosis and recent trend toward improved survival. Ann Oncol (2008) 19(10):1727–33. doi: 10.1093/annonc/mdn351 PMC2735065 18515795

[2] Fraenkel M Kim M Faggiano A de Herder WW Valk GD Knowledge N. Incidence of gastroenteropancreatic neuroendocrine tumours: A systematic review of the literature. Endocr Relat Cancer (2014) 21(3):R153–63. doi: 10.1530/ERC-13-0125 24322304

[3] Dasari A Shen C Halperin D Zhao B Zhou S Xu Y. Trends in the incidence, prevalence, and survival outcomes in patients with neuroendocrine tumors in the united states. JAMA Oncol (2017) 3(10):1335–42. doi: 10.1001/jamaoncol.2017.0589 PMC5824320 28448665

[4] Cives M Strosberg JR. Gastroenteropancreatic neuroendocrine tumors. CA Cancer J Clin (2018) 68(6):471–87. doi: 10.3322/caac.21493 30295930

[5] Niederle MB Hackl M Kaserer K Niederle B. Gastroenteropancreatic neuroendocrine tumours: The current incidence and staging based on the who and European neuroendocrine tumour society classification: An analysis based on prospectively collected parameters. Endocr Relat Cancer (2010) 17(4):909–18. doi: 10.1677/ERC-10-0152 20702725

[6] Nigri G Petrucciani N Debs T Mangogna LM Crovetto A Moschetta G. Treatment options for pnet liver metastases: A systematic review. World J Surg Oncol (2018) 16(1):142. doi: 10.1186/s12957-018-1446-y 30007406 PMC6046097

[7] Yao JC Eisner MP Leary C Dagohoy C Phan A Rashid A. Population-based study of islet cell carcinoma. Ann Surg Oncol (2007) 14(12):3492–500. doi: 10.1245/s10434-007-9566-6 PMC2077912 17896148

[8] Rossi RE Massironi S Conte D Peracchi M. Therapy for metastatic pancreatic neuroendocrine tumors. Ann Transl Med (2014) 2(1):8. doi: 10.3978/j.issn.2305-5839.2013.03.01 25332984 PMC4200651

[9] Yao JC Pavel M Lombard-Bohas C Van Cutsem E Voi M Brandt U. Everolimus for the treatment of advanced pancreatic neuroendocrine tumors: Overall survival and circulating biomarkers from the randomized, phase iii radiant-3 study. J Clin Oncol (2016) 34(32):3906–13. doi: 10.1200/JCO.2016.68.0702 PMC5791842 27621394

[10] Faivre S Niccoli P Castellano D Valle JW Hammel P Raoul JL. Sunitinib in pancreatic neuroendocrine tumors: Updated progression-free survival and final overall survival from a phase iii randomized study. Ann Oncol (2017) 28(2):339–43. doi: 10.1093/annonc/mdw561 27836885

[11] Ma ZY Gong YF Zhuang HK Zhou ZX Huang SZ Zou YP. Pancreatic neuroendocrine tumors: A review of serum biomarkers, staging, and management. World J Gastroenterol (2020) 26(19):2305–22. doi: 10.3748/wjg.v26.i19.2305 PMC7243647 32476795

[12] Brabander T van der Zwan WA Teunissen JJM Kam BLR Feelders RA de Herder WW. Long-term efficacy, survival, and safety of [(177)Lu-Dota(0),Tyr(3)]Octreotate in patients with gastroenteropancreatic and bronchial neuroendocrine tumors. Clin Cancer Res (2017) 23(16):4617–24. doi: 10.1158/1078-0432.CCR-16-2743 28428192

[13] Kloppel G La Rosa S. Ki67 labeling index: Assessment and prognostic role in gastroenteropancreatic neuroendocrine neoplasms. Virchows Arch (2018) 472(3):341–9. doi: 10.1007/s00428-017-2258-0 29134440

[14] Rindi G Kloppel G Alhman H Caplin M Couvelard A de Herder WW. Tnm staging of foregut (Neuro)Endocrine tumors: A consensus proposal including a grading system. Virchows Arch (2006) 449(4):395–401. doi: 10.1007/s00428-006-0250-1 16967267 PMC1888719

[15] Rindi G Wiedenmann B. Neuroendocrine neoplasms of the gut and pancreas: New insights. Nat Rev Endocrinol (2011) 8(1):54–64. doi: 10.1038/nrendo.2011.120 21808296

[16] Khan MS Luong TV Watkins J Toumpanakis C Caplin ME Meyer T. A comparison of ki-67 and mitotic count as prognostic markers for metastatic pancreatic and midgut neuroendocrine neoplasms. Br J Cancer (2013) 108(9):1838–45. doi: 10.1038/bjc.2013.156 PMC3658531 23579216

[17] Scoazec J-Y Couvelard A Reseau T. Classification of pancreatic neuroendocrine tumours: Changes made in the 2017 who classification of tumours of endocrine organs and perspectives for the future. ANNALES PATHOLOGIE (2017) 37(6):444–56. doi: 10.1016/j.annpat.2017.10.003 29169836

[18] Nagtegaal ID Odze RD Klimstra D Paradis V Rugge M Schirmacher P. The 2019 who classification of tumours of the digestive system. Histopathology (2020) 76(2):182–8. doi: 10.1111/his.13975 PMC7003895 31433515

[19] Oberg K Knigge U Kwekkeboom D Perren A Group EGW. Neuroendocrine gastro-Entero-Pancreatic tumors: Esmo clinical practice guidelines for diagnosis, treatment and follow-up. Ann Oncol (2012) 23 Suppl 7:vii124–30. doi: 10.1093/annonc/mds295 22997445

[20] Grillo F Albertelli M Brisigotti MP Borra T Boschetti M Fiocca R. Grade increases in gastroenteropancreatic neuroendocrine tumor metastases compared to the primary tumor. NEUROENDOCRINOLOGY (2016) 103(5):452–9. doi: 10.1159/000439434 26337010

[21] Keck KJ Choi A Maxwell JE Li G O'Dorisio TM Breheny P. Increased grade in neuroendocrine tumor metastases negatively impacts survival. Ann OF Surg Oncol (2017) 24(8):2206–12. doi: 10.1245/s10434-017-5899-y PMC5772651 28560597

[22] Botling J Lamarca A Bajic D Norlen O Lonngren V Kjaer J. High-grade progression confers poor survival in pancreatic neuroendocrine tumors. NEUROENDOCRINOLOGY (2020) 110(11-12):891–8. doi: 10.1159/000504392 31658459

[23] Pavel M Baudin E Couvelard A Krenning E Oberg K Steinmueller T. Enets consensus guidelines for the management of patients with liver and other distant metastases from neuroendocrine neoplasms of foregut, midgut, hindgut, and unknown primary. NEUROENDOCRINOLOGY (2012) 95(2):157–76. doi: 10.1159/000335597 22262022

[24] Wang WQ Liu L Xu HX Wu CT Xiang JF Xu J. Infiltrating immune cells and gene mutations in pancreatic ductal adenocarcinoma. Br J Surg (2016) 103(9):1189–99. doi: 10.1002/bjs.10187 27256393

[25] Zhang W-H Wang W-Q Han X Gao H-L Xu S-S Li S. Infiltrating pattern and prognostic value of tertiary lymphoid structures in resected non-functional pancreatic neuroendocrine tumors. J FOR IMMUNOTHERAPY OF Cancer (2020) 8(2):e001188. doi: 10.1136/jitc-2020-001188 PMC7559054 33055204

[26] Zhang WH Wang WQ Gao HL Xu SS Li S Li TJ. Tumor-infiltrating neutrophils predict poor survival of non-functional pancreatic neuroendocrine tumor. J Clin Endocrinol Metab (2020) 105(7):dgaa196. doi: 10.1210/clinem/dgaa196 32285127

[27] Wang WQ Zhang WH Gao HL Huang D Xu HX Li S. A novel risk factor panel predicts early recurrence in resected pancreatic neuroendocrine tumors. J Gastroenterol (2021) 56(4):395–405. doi: 10.1007/s00535-021-01777-0 33742253

[28] Zhang WH Gao HL Liu WS Qin Y Ye Z Lou X. Supplementary material for the manuscript “A real-life treatment cohort of pancreatic neuroendocrine tumors: High-grade increase in metastases confers poor survival. (2002) figshare. doi: 10.6084/m9.figshare.19350545.v8 PMC9399842 36034463

[29] Tanaka M Shinozaki-Ushiku A Kunita A Yasunaga Y Akamatsu N Hasegawa K. High-grade transformation of pancreatic neuroendocrine tumor associated with Tp53 mutations: A diagnostic pitfall mimicking neuroendocrine carcinoma. Pathol Int (2022). doi: 10.1111/pin.13252 35698921

[30] Ali AS Gronberg M Federspiel B Scoazec J-Y Hjortland GO Gronbaek H. Expression of P53 protein in high-grade gastroenteropancreatic neuroendocrine carcinoma. PloS One (2017) 12(11):e0187667. doi: 10.1371/journal.pone.0187667 29112960 PMC5675414

[31] Alexandraki KI Kaltsatou M Kyriakopoulos G Mavroeidi V Kostopoulou A Atlan K. Distinctive features of pancreatic neuroendocrine neoplasms exhibiting an increment in proliferative activity during the course of the disease. Endocrine (2021) 72(1):279–86. doi: 10.1007/s12020-020-02540-w 33175321

[32] Konukiewitz B Schlitter AM Jesinghaus M Pfister D Steiger K Segler A. Somatostatin receptor expression related to Tp53 and Rb1 alterations in pancreatic and extrapancreatic neuroendocrine neoplasms with a Ki67-index above 20%. MODERN Pathol (2017) 30(4):587–98. doi: 10.1038/modpathol.2016.217 28059098

[33] Perysinakis I Aggeli C Kaltsas G Zografos GN. Neoadjuvant therapy for advanced pancreatic neuroendocrine tumors: An emerging treatment modality? Hormones (Athens) (2016) 15(1):15–22. doi: 10.14310/horm.2002.1636 26732156

[34] Lania A Ferrau F Rubino M Modica R Colao A Faggiano A. Neoadjuvant therapy for neuroendocrine neoplasms: Recent progresses and future approaches. Front Endocrinol (Lausanne) (2021) 12:651438. doi: 10.3389/fendo.2021.651438 34381421 PMC8350565

[35] Shi H Zhang Q Han C Zhen D Lin R. Variability of the ki-67 proliferation index in gastroenteropancreatic neuroendocrine neoplasms - a single-center retrospective study. BMC ENDOCRINE Disord (2018) 18:51–7. doi: 10.1186/s12902-018-0274-y PMC6064167 30055596

[36] Furukawa T Ozaka M Takamatsu M Takazawa Y Inamura K Inoue Y. Ki-67 labeling index variability between surgically resected primary and metastatic hepatic lesions of gastroenteropancreatic neuroendocrine neoplasms. Int J OF Surg Pathol (2021) 29(5):475–81. doi: 10.1177/1066896921990715 33543658

[37] Miller HC Drymousis P Flora R Goldin R Spalding D Frilling A. Role of ki-67 proliferation index in the assessment of patients with neuroendocrine neoplasias regarding the stage of disease. World J OF Surg (2014) 38(6):1353–61. doi: 10.1007/s00268-014-2451-0 24493070

[38] Dumars C Foubert F Touchefeu Y Regenet N Senellart H Matysiak-Budnik T. Can Pph3 be helpful to assess the discordant grade in primary and metastatic enteropancreatic neuroendocrine tumors? ENDOCRINE (2016) 53(2):395–401. doi: 10.1007/s12020-016-0944-3 27048356

[39] Adesoye T Daleo MA Loeffler AG Winslow ER Weber SM Cho CS. Discordance of histologic grade between primary and metastatic neuroendocrine carcinomas. Ann OF Surg Oncol (2015) 22:S817–S21. doi: 10.1245/s10434-015-4733-7 PMC4859783 26193965

[40] Zen Y Heaton N. Elevated ki-67 labeling index in 'Synchronous liver metastases' of well differentiated enteropancreatic neuroendocrine tumor. Pathol Int (2013) 63(11):532–8. doi: 10.1111/pin.12108 24274715

[41] Holmager P Langer SW Federspiel B Willemoe GL Garbyal RS Melchior L. Increase of ki-67 index and influence on mortality in patients with neuroendocrine neoplasms. J OF Neuroendocrinol (2021) 33(9):e13018. doi: 10.1111/jne.13018 34414612

[42] Panzuto F Cicchese N Partelli S Rinzivillo M Capurso G Merola E. Impact of Ki67 re-assessmentat time of disease progression in patients with pancreatic neuroendocrine neoplasms. PloS One (2017) 12(6):e0179445. doi: 10.1371/journal.pone.0179445 28644861 PMC5482443

[43] Cicchese N Panzuto F Rinzivillo M Capurso G Merola E Pucci E. Reassessment of proliferative activity at disease progression in neuroendocrine neoplasms. GASTROENTEROLOGY (2016) 150(4):S301–S. doi: 10.1016/S0016-5085(16)31054-X

[44] Singh S Hallet J Rowsell C Law CHL. Variability of Ki67 labeling index in multiple neuroendocrine tumors specimens over the course of the disease. EJSO (2014) 40(11):1517–22. doi: 10.1016/j.ejso.2014.06.016 25088936

[45] Shi CJ Gonzalez RS Zhao ZG Koyama T Cornish TC Hande KR. Liver metastases of small intestine neuroendocrine tumors ki-67 heterogeneity and world health organization grade discordance with primary tumors. Am J OF Clin Pathol (2015) 143(3):398–404. doi: 10.1309/AJCPQ55SKOCYFZHN 25696798 PMC4354931

[46] Richards-Taylor S Tilley C Jaynes E Hu H Armstrong T Pearce NW. Clinically significant differences in ki-67 proliferation index between primary and metastases in resected pancreatic neuroendocrine tumors. PANCREAS (2017) 46(10):1354–8. doi: 10.1097/MPA.0000000000000933 28984786

[47] Alcala N Leblay N Gabriel AAG Mangiante L Hervas D Giffon T. Integrative and comparative genomic analyses identify clinically relevant pulmonary carcinoid groups and unveil the supra-carcinoids. Nat Commun (2019) 10:3407–27. doi: 10.1038/s41467-019-11276-9 PMC6702229 31431620

[48] Dinter H Bohnenberger H Beck J Bornemann-Kolatzki K Schutz E Kuffer S. Molecular classification of neuroendocrine tumors of the thymus. J OF Thorac Oncol (2019) 14(8):1472–83. doi: 10.1016/j.jtho.2019.04.015 31042566

[49] Pelosi G Bianchi F Dama E Metovic J Barella M Sonzogni A. A subset of Large cell neuroendocrine carcinomas in the gastroenteropancreatic tract may evolve from pre-existing well-differentiated neuroendocrine tumors. ENDOCRINE Pathol (2021) 32(3):396–407. doi: 10.1007/s12022-020-09659-6 33433886

[50] Pelosi G Bianchi F Hofman P Pattini L Strobel P Calabrese F. Recent advances in the molecular landscape of lung neuroendocrine tumors. Expert Rev OF Mol DIAGNOSTICS (2019) 19(4):281–97. doi: 10.1080/14737159.2019.1595593 30900485

[51] Tang LH Untch BR Reidy DL O'Reilly E Dhall D Jih L. Well-differentiated neuroendocrine tumors with a morphologically apparent high-grade component: A pathway distinct from poorly differentiated neuroendocrine carcinomas. Clin Cancer Res (2016) 22(4):1011–7. doi: 10.1158/1078-0432.CCR-15-0548 PMC4988130 26482044

[52] Pelosi G Bresaola E Bogina G Pasini F Rodella S Castelli P. Endocrine tumors of the pancreas: Ki-67 immunoreactivity on paraffin sections is an independent predictor for malignancy: A comparative study with proliferating-cell nuclear antigen and progesterone receptor protein immunostaining, mitotic index, and other clinicopathologic variables. Hum Pathol (1996) 27(11):1124–34. doi: 10.1016/S0046-8177(96)90303-2 8912819

[53] Partelli S Inama M Rinke A Begum N Valente R Fendrich V. Long-term outcomes of surgical management of pancreatic neuroendocrine tumors with synchronous liver metastases. Neuroendocrinology (2015) 102(1-2):68–76. doi: 10.1159/000431379 26043944

