# Supplementary material for: A real-life treatment cohort of pancreatic neuroendocrine tumors: High-grade increase in metastases confers poor survival
Source: Front Endocrinol (Lausanne). 2022 Aug 10;13:941210. doi: 10.3389/fendo.2022.941210 (PMC9399842; doi:10.3389/fendo.2022.941210)
Supplement: Supplementary file 1 [file DataSheet_1.docx]

Supplementary Material

# Supplementary Data

## Supplementary methods

### Immunohistochemistry and re-evaluation

Briefly, 4 µm thick FFPE sections were deparaffinized and rehydrated. Then, antigen retrieval was performed in pH 6.0 citrate buffer using a microwave oven for 15 min. Blocking of nonspecific binding was performed by incubation with 5% goat serum at room temperature. Sections were incubated with anti-Ki67 antibody (MIB-1, Dako Corporation, CA, USA), and secondary antibody was then applied. The slides were then counterstained with hematoxylin and cover-slipped. In cases without available tumor samples, the Ki67 index was obtained from the pathology report issued by the pathology department of the FUSCC.

The slides were independently reviewed by two observers (Dr. Wu-Hu Zhang and Dr. Zeng Ye) to determine the Ki67 labeling index. A total of 500–2000 tumor cells with the highest immunostaining per 400× high-power fields (HPFs) were examined, and the percentage of Ki67-positive nuclei staining was reported. These re-evaluated Ki67 values were comparable to those originally provided by the pathology department. The Ki67 values of 9 patients who underwent needle biopsy were also provided by the pathology department, and although they were not as accurate as those of resection samples, they could serve as an important reference.

## Supplementary results

### Subgroup Analysis of PanNETs with High-grade Increase in Metastases

Logistic regression models were used to control confounding. The results showed that confounding factors, including sex, age, tumor size, location, grade at first diagnosis, surgery and chemotherapy, did not show any significant difference between the groups with or without a high-grade increase in metastases (Supplementary **Table S4**).

### Estimating Impact of Selection Bias

To assess the extent of selection bias, another group of patients called the “bias-control group” was identified among patients without eligibility, i.e., sporadic PanNET cases lacking paired primary tumors and metastases with an available Ki67 index (Supplementary **Figure** **S3**). To reduce clinical heterogeneity, this comparison was made only for patients with AJCC 8^th^ stage IV and G1/G2 at first diagnosis, and 67 patients remained in the study cohort and 33 remained in the bias-control group. Survival did not show a significant difference between the 2 groups: the median PFS was 12.0 (95% CI: 8.15–15.85) months in the selected study cohort and 11.0 (95% CI: 7.72-14.29) months in the selected bias-control group; Cox regression HR 1.235 (95% CI 0.771-1.977), *P*=0.380. The mean OS was 84.4 (95% CI 76.23-92.56) months in the selected study cohort and 62.0 (95% CI 49.66-74.38) months in the selected bias-control group; Cox regression HR 2.113 (95% CI 0.813-5.495), *P*=0.125 (Supplementary **Figure S4)**.

# Supplementary Figures and Tables

## Supplementary Figures

**
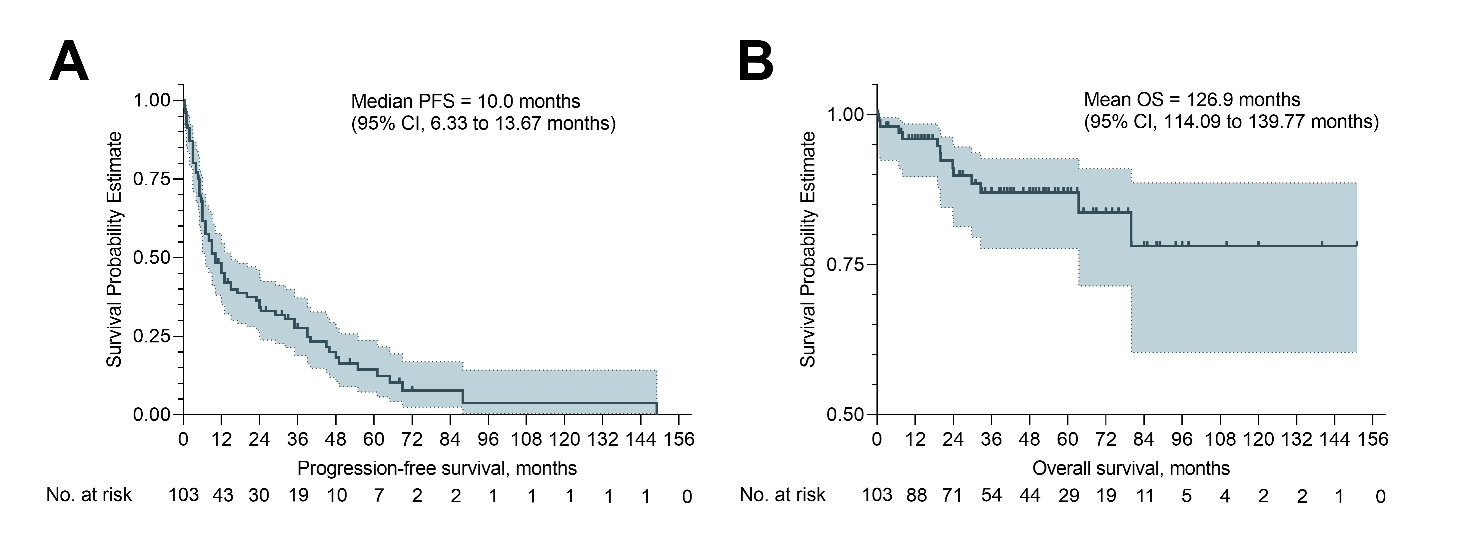
**

**Figure S1.** Kaplan-Meier progression-free (A) and overall survival (B) curves of the study cohort. The shaded area represents the 95% confidence interval (CI).

**
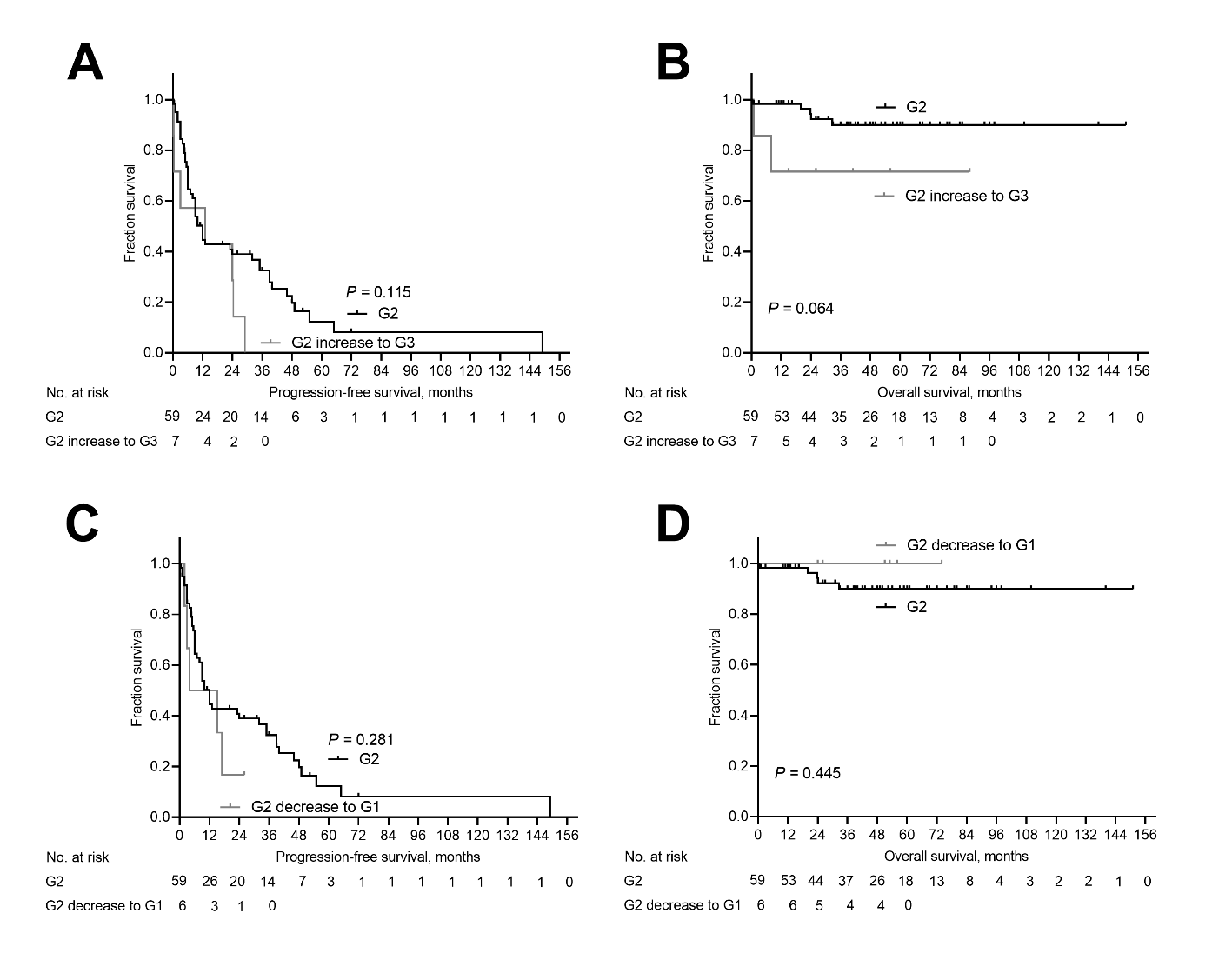
Figure S2.** Kaplan-Meier curves depicting progression-free survival (PFS) and overall survival (OS) for patients with and without grade changes.

(A) No statistically significant difference in PFS (*P*=0.115) were shown between patients with G2 increase to G3 and patients with stable G2, although patients with G2 increase to G3 had decreased PFS.

(B) No statistically significant difference in OS (*P*=0.064) were shown between patients with G2 increase to G3 and patients with stable G2, although patients with G2 increase to G3 had decreased OS.

(C) No statistically significant difference in PFS (*P*=0.281) were shown between patients with G2 decrease to G1 and patients with stable G2.

(D) No statistically significant difference in OS (*P*=0.445) were shown between patients with G2 decrease to G1 and patients with stable G2, although patients with G2 decrease to G1 had longer OS.

**
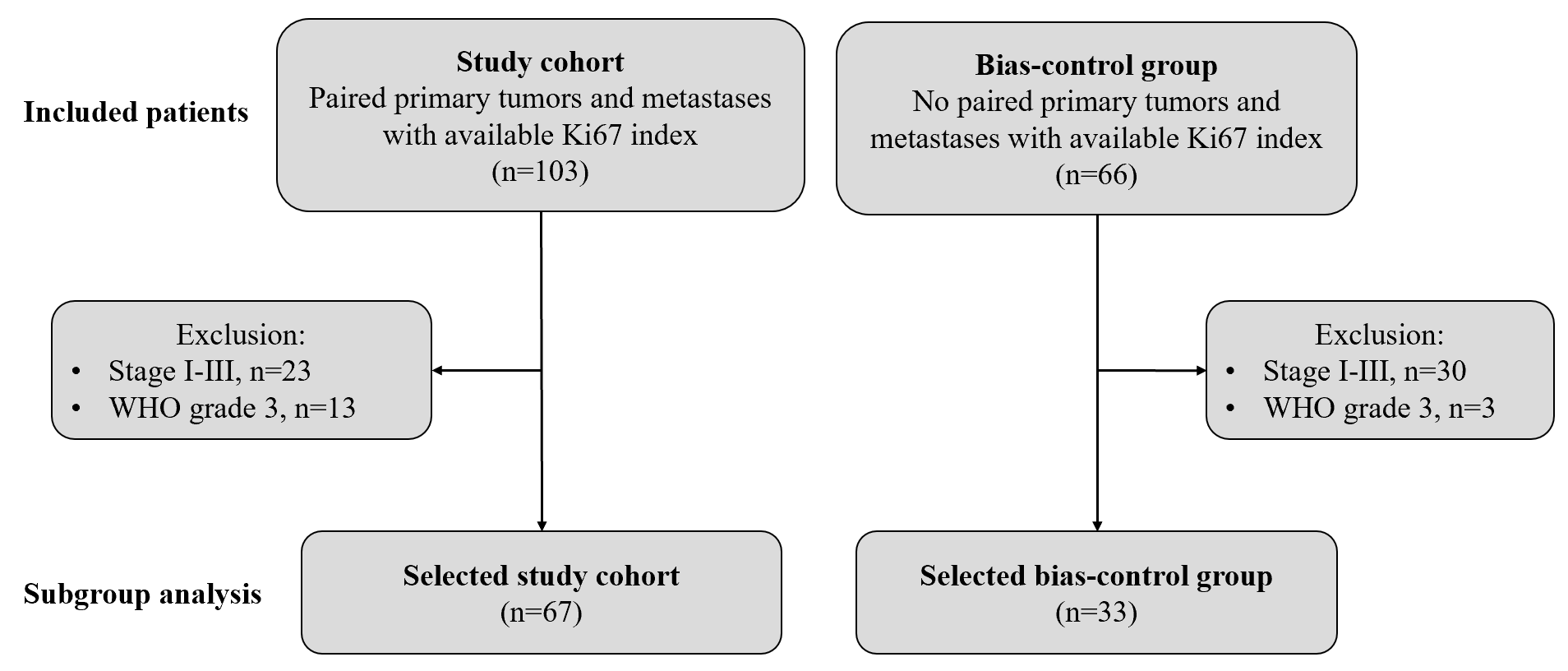
Figure S3.** Assessment of selection bias through comparison of the study cohort to a bias–control group. In the study cohort, patients with grade 1–2 and stage IV at first diagnosis were selected for analysis, and 67 patients remained. In the bias control group, 33 were excluded and 33 patients remained.


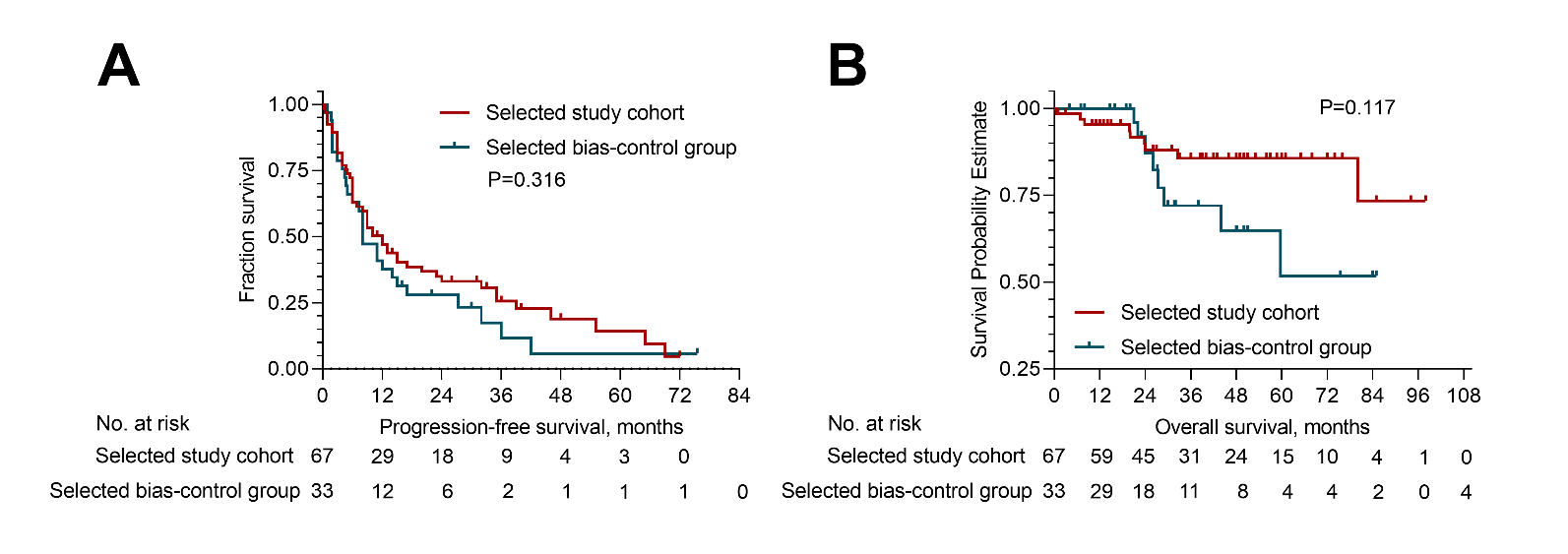
**Figure S4.** Kaplan-Meier progression-free (PFS) and overall survival (OS) curves of the selected study cohort and selected bias-control group.

(A) The median PFS of the selected study cohort was 12.0 (95% CI 8.15–15.85) months and was 11.0 (95% CI 7.72-14.29) month in the selected bias-control group, log-rank *P*=0.316. Cox regression HR 1.235 (95% CI 0.771-1.977), *P*=0.380.

(B) The mean OS was 84.4 (95% CI 76.23-92.56) months in the selected study cohort and 62.0 (95% CI 49.66-74.38) months in the selected bias-control group, log-rank *P*=0.117. Cox regression HR 2.113 (95% CI 0.813-5.495), *P*=0.125.

**
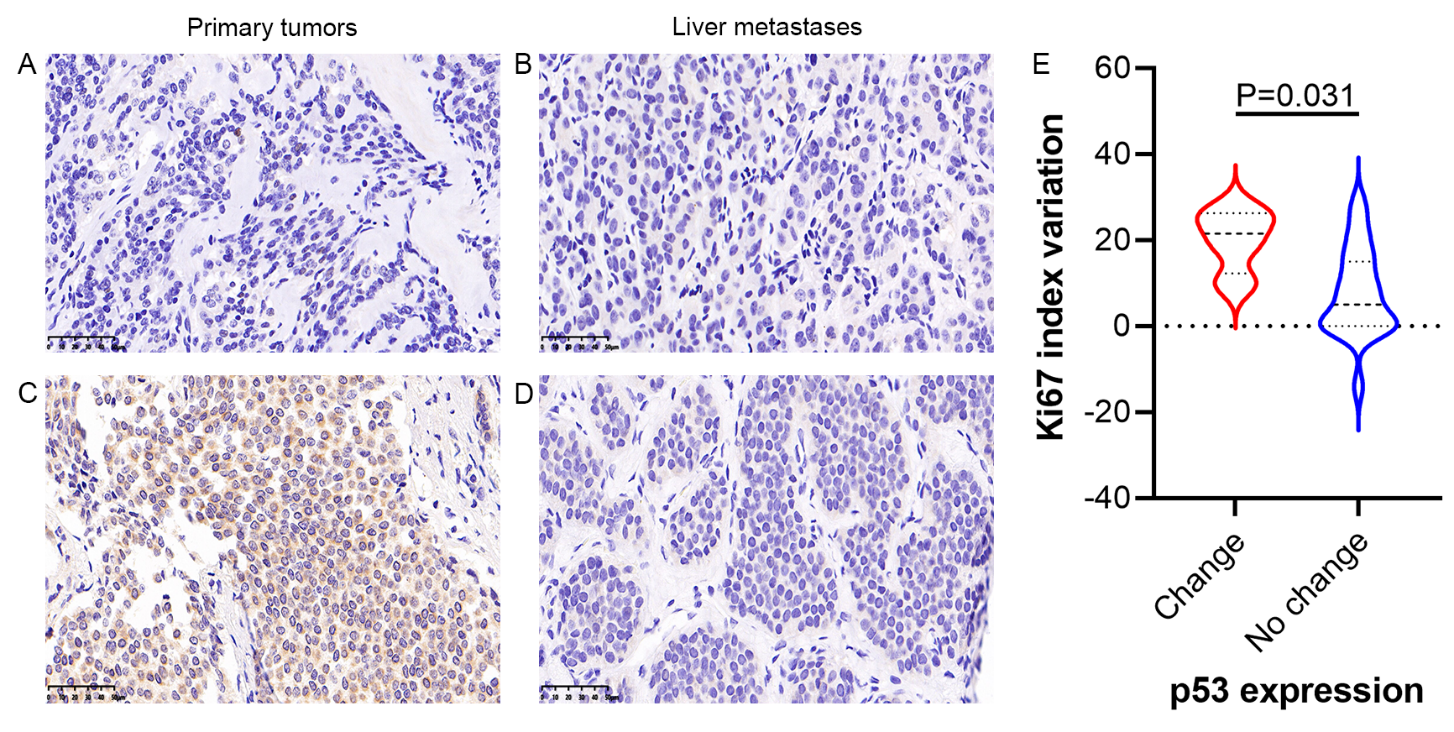
Figure S5.** Representative images of changes in p53 expression in liver metastases compared to primary tumors.

(A-B) A wild-type pattern of p53 expression in primary tumors (A), and complete absence in liver metastases (B).

(C-D) Positive p53 expression in primary tumors (C), and complete absence in liver metastases (D).

(E) Group with changes in p53 expression exhibited higher Ki67 variation than group with no change in p53 expression.

## Supplementary Tables

**Table S1** Specific information of the four patients with functional PanNETs

| **Characteristic** | **Patient 1** | **Patient 2** | **Patient 3** | **Patient 4** |
| --- | --- | --- | --- | --- |
| Gender | Female | Female | Female | male |
| Age, years | 35 | 29 | 32 | 50 |
| Tumor size, mm | 19 | 20 | 40 | 50 |
| Location | Body | Tail | Body | Tail |
| Types of functional PanNETs | Insulinoma | Insulinoma | Insulinoma | Glucagonoma |
| Lymph node positive | Yes | NA | Yes | No |
| Perineural invasion | No | NA | Yes | No |
| Microvascular invasion | No | NA | Yes | Yes |
| CgA**^a^** | Positive | Positive | Positive | Positive |
| Syn**^a^** | Positive | Positive | Positive | Positive |
| DAXX**^a^** | NA | NA | Positive | NA |
| ATRX**^a^** | NA | NA | Positive | NA |
| SSTR**^a^** | NA | NA | SSTR2 Positive | SSTR2 Positive |
| NSE classification | Low | Low | High | Low |
| PROGRP classification | Low | NA | NA | Low |
| Metastases site | Liver | Liver | Liver | Nodal |
| Metastases type | Synchronous | Metachronous | Synchronous | Metachronous |
| AJCC^8th^ TNM stage | IV | II | IV | II |
| WHO classification**^b^** | G2 | G1 | G2 | G1 |
| Ki67 index variation**^c^** | 0 | 0 | 0 | +4 |
| Grade changes**^c^** | Stable | Stable | Stable | G1 increase to G2 |
| Neoadjuvant treatment | No | No | No | No |
| Operating methods | Distal pancreatectomy with LM resection | Distal pancreatectomy | Distal pancreatectomy with LM resection | Distal pancreatectomy |
| Adjuvant treatment | SSAs | No | SSAs+CAPTEM | No |
| Note: PanNETs: pancreatic neuroendocrine tumors; CgA: chromogranin; Syn: synaptophysin; DAXX: death domain associated protein; ATRX: alpha-thalassemia/mental retardation X-linked; SSTR: somatostatin receptor; NSE: neuron specific enolase; PROGRP: progastrin releasing peptide; AJCC: American Joint Committee on Cancer; WHO: World Health Organization; LM: liver metastases; SSAs: somatostatin analogs; CAPTEM: capecitabine and temozolomide; NA: not available; **^a^**The expression in primary tumors; **^b^**Grade at first diagnosis; **^c^**Ki67 index in metastases compared to primary tumors. | | | | |

**Table S2** Specific information of the eleven patients with G1/G2 increase to G3

| **Characteristic** | **Patient 1** | **Patient 2** | **Patient 3** | **Patient 4** | **Patient 5** | **Patient 6** | **Patient 7** | **Patient 8** | **Patient 9** | **Patient 10** | **Patient 11** |
| --- | --- | --- | --- | --- | --- | --- | --- | --- | --- | --- | --- |
| Gender | Male | Female | Male | Female | Male | Female | Female | Female | Female | Male | Female |
| Age, years | 43 | 31 | 67 | 39 | 72 | 45 | 44 | 54 | 30 | 50 | 54 |
| Tumor size, mm | 32 | 60 | 25 | 45 | 30 | 90 | 65 | 30 | 20 | 30 | 30 |
| Location | Body | Head | Head | Head | Head | Body | Body | Tail | Body | Body | Tail |
| Lymph node positive | Yes | No | No | No | No | Yes | Yes | NA | Yes | No | NA |
| Perineural invasion | Yes | No | Yes | NA | No | Yes | Yes | NA | Yes | No | NA |
| Microvascular invasion | Yes | No | Yes | NA | Yes | Yes | Yes | NA | Yes | Yes | NA |
| CgA**^a^** | Positive | NA | Positive | Positive | Positive | Positive | Positive | Positive | Positive | Positive | Positive |
| Syn**^a^** | Positive | NA | Positive | Positive | Positive | Positive | Positive | Positive | Positive | Positive | Positive |
| DAXX**^a^** | NA | NA | Positive | NA | NA | Positive | Positive | NA | Positive | Positive | NA |
| ATRX**^a^** | NA | NA | Positive | NA | NA | Positive | Positive | NA | Positive | Positive | NA |
| SSTR**^a^** | NA | NA | SSTR2 and 5 positive | SSTR2 positive | SSTR2 and 5 positive | SSTR2 and 5 positive | SSTR2 and 5 positive | NA | SSTR2 positive | SSTR2 and 5 positive | NA |
| NSE classification | Low | Low | Low | High | Low | High | High | High | Low | Low | High |
| PROGRP classification | NA | NA | Low | NA | Low | NA | NA | Low | Low | NA | NA |
| Metastases site | Liver | Liver | Liver | Liver | Liver | Liver | Liver | Liver | Liver | Liver | Liver |
| Metastases type | Synchronous | Metachronous | Metachronous | Metachronous | Metachronous | Synchronous | Synchronous | Synchronous | Synchronous | Synchronous | Synchronous |
| AJCC^8th^ TNM stage | IV | II | II | II | II | IV | IV | IV | IV | IV | IV |
| WHO classification**^b^** | G1 | G2 | G2 | G2 | G2 | G3 | G3 | G2 | G3 | G1 | G2 |
| Ki67 index variation**^c^** | +24 | +15 | +15 | +22 | +10 | +24 | +26 | +13 | +20 | +29 | +27 |
| Grade changes | G1 increase to G3 | G2 increase to G3 | G2 increase to G3 | G2 increase to G3 | G2 increase to G3 | G1 increase to G3 | G2 increase to G3 | G2 increase to G3 | G1 increase to G3 | G1 increase to G3 | G2 increase to G3 |
| Neoadjuvant treatment | No | No | No | No | No | CAPTEM with or without targeted therapy | CAPTEM with or without targeted therapy | NA | SSAs+CAPTEM with or without targeted therapy | Other chemotherapy | NA |
| Operating methods | Distal pancreatectomy with LM resection | Pancreatoduodenectomy | Pancreatoduodenectomy | Distal pancreatectomy | Pancreatoduodenectomy | Distal pancreatectomy with LM resection | Distal pancreatectomy with LM resection | Needle biopsy | Pancreatoduodenectomy with LM resection | Pancreatoduodenectomy with LM resection | Needle biopsy |
| Adjuvant treatment | SSAs | SSAs | SSAs+CAPTEM with or without targeted therapy | SSAs | SSAs+CAPTEM with or without targeted therapy | Targeted therapy | NA | NA | NA | Locoregional treatment | NA |
| Treatment for patients with biopsy | NA | NA | NA | NA | NA | NA | NA | Targeted therapy | NA | NA | SSAs |
| Note: CgA: chromogranin; Syn: synaptophysin; DAXX: death domain associated protein; ATRX: alpha-thalassemia/mental retardation X-linked; SSTR: somatostatin receptor; NSE: neuron specific enolase; PROGRP: progastrin releasing peptide; AJCC: American Joint Committee on Cancer; WHO: World Health Organization; LM: liver metastases; SSAs: somatostatin analogs; CAPTEM: capecitabine and temozolomide; NA: not available; **^a^**The expression in primary tumors; **^b^**Grade at first diagnosis; **^c^**Ki67 index in metastases compared to primary tumors. | | | | | | | | | | | |

**Table S3** Demographics and clinical characteristics of the advanced and resected patients with NAT versus patients without NAT

| **Characteristic** | **NAT**  **(n=31)** | **Not NAT**  **(n=37)** | ***P*-value** |
| --- | --- | --- | --- |
|  | No. (%) | No. (%) |  |
| **Gender** |  |  | 0.361 |
| Male | 16 (51.6) | 15 (40.5) |  |
| Female | 15 (48.4) | 22 (59.5) |  |
| **Age, years, median** | 50.0 | 51.0 | 0.438 |
| **Tumor size, mm** |  |  | 0.072 |
| Mean (SD) | 50.4 (3.6) | 48.2 (2.5) |  |
| Median (range) | 35.0 (8.0-160.0) | 44.0 (12.0-140.0) |  |
| **Location** |  |  | 0.279 |
| Head | 7 (22.6) | 7 (18.9) |  |
| Neck | 2 (6.5) | 0 (0.0) |  |
| Body | 3 (9.7) | 9 (24.3) |  |
| Tail | 7 (22.6) | 10 (27.0) |  |
| Body-Tail | 12 (38.7) | 11 (29.7) |  |
| **Functional** |  |  | 0.189 |
| Yes | 0 (0.0) | 2 (5.4) |  |
| No | 31 (100.0) | 35 (94.6) |  |
| **Lymph node positive** |  | n=35 | 0.159 |
| Yes | 16 (51.6) | 24 (68.6) |  |
| No | 15 (48.4) | 11 (31.4) |  |
| **Perineural invasion** |  | n=35 | 0.805 |
| Yes | 15 (48.4) | 18 (51.4) |  |
| No | 16 (51.6) | 17 (48.6) |  |
| **Microvascular invasion** |  | n=35 | 0.727 |
| Yes | 20 (64.5) | 24 (68.6) |  |
| No | 11 (35.5) | 11 (31.4) |  |
| **Metastases** |  |  |  |
| Site |  |  | 0.356 |
| Liver | 31 (100.0) | 36 (97.3) |  |
| Peritoneum/others | 0 (0.0) | 1 (2.3) |  |
| Type of LM |  | n=36 | **0.009** |
| I | 2 (6.5) | 6 (16.7) |  |
| II | 2 (6.5) | 12 (33.3) |  |
| III | 27 (87.1) | 18 (50.0) |  |
| **WHO classification^a^** |  |  | 0.538 |
| G1 | 2 (6.5) | 4 (10.8) |  |
| G2 | 23 (74.2) | 29 (78.4) |  |
| G3 | 6 (19.4) | 4 (10.8) |  |
| **Operating methods** |  |  | 0.352 |
| Total pancreatectomy with LM resection  Middle pancreatectomy with LM resection  Pancreatoduodenectomy with LM resection  Distal Pancreatectomy with LM resection | 0 (0.0)  0 (0.0)  8 (25.8)  23 (74.2) | 2 (5.4)  1 (2.7)  6 (16.2)  28 (75.7) |  |
| **Treatment** |  |  | NA |
| Neoadjuvant treatment  SSAs with or without targeted therapy  CAPTEM with or without targeted therapy  SSAs+CAPTEM with or without targeted therapy  Locoregional treatment  Other chemotherapy  Targeted therapy  Others | 9 (29.0)  11 (35.5)  7 (22.6)  1 (3.2)  2 (6.5)  1 (3.2)  0 (0.0) | n=0 |  |
| Adjuvant treatment  SSAs with or without targeted therapy  CAPTEM with or without targeted therapy  SSAs+CAPTEM with or without targeted therapy  Locoregional treatment  Other chemotherapy  Targeted therapy | n=27  12 (44.4)  3 (11.1)  1 (3.7)  5 (18.5)  2 (7.4)  4 (14.8) | n=34  13 (38.2)  1 (2.9)  3 (8.8)  6 (17.6)  2 (5.9)  9 (26.5) | 0.130 |
| **Ki67 index variation^b^** |  |  | 0.798 |
| **Grade changes^c^**  Increase  Stable or decrease | 8 (25.8)  23 (74.2) | 2 (5.4)  35 (94.6) | **0.018** |
| Note: PanNETs: pancreatic neuroendocrine tumors; NAT: neoadjuvant treatment; LM: liver metastases; WHO: World Health Organization; SSAs: somatostatin analogs; CAPTEM: capecitabine and temozolomide; NA: not available; **^a^**Grade at first diagnosis; **^b^**Ki67 index variation was continuous variables, and were compared by non-parametric tests; **^c^**grade in metastases compared to primary tumors; bold values indicate statistical significance. | | | |

**Table S4** Demographics and clinical characteristics in patients with high-grade increase in metastases versus patients without WHO high-grade increase in metastases

| **Characteristic** | **No high-grade increase in metastases**  **(n=92)** | **High-grade increase in metastases (n=11)** | **Prediction of high-grade increase in metastases (logistic regression)** | | **Multivariable logistic regression** | |
| --- | --- | --- | --- | --- | --- | --- |
|  | No. (%) | No. (%) | OR, 95% CI | *P*-value | OR, 95% CI | *P*-value |
| **Gender** |  |  |  | 0.397 |  |  |
| Male | 46 (50.0) | 4 (36.4) | 1 (Ref) |  |  |  |
| Female | 46 (50.0) | 7 (63.6) | 1.750 (0.479-6.387) |  |  |  |
| **Age, years, median (range)** | 51.5 (18-81) | 45.0 (30-72) | 0.983 (0.936-1.034) | 0.510 |  |  |
| **Tumor size, mm, median (range)** | 40.0 (8.0-160.0) | 31.0 (20.0-90.0) | 0.914 (0.703-1.189) | 0.503 |  |  |
| **Location** |  |  |  | 0.160 |  | 0.277 |
| Head | 23 (25.0) | 5 (45.5) | 1 (Ref) |  | 1 (Ref) |  |
| Neck, body and tail | 69 (75.0) | 6 (54.5) | 2.500 (0.697-8.966) |  | 2.088 (0.554-7.869) |  |
| **WHO classification^a^** |  |  |  |  |  |  |
| G1 | 8 (8.7) | 2 (18.2) | 1 (Ref) |  | 1 (Ref) |  |
| G2 | 70 (76.1) | 6 (54.4) | 0.343 (0.059-1.992) | 0.233 | 0.393 (0.066-2.361) | 0.308 |
| G3 | 14 (15.2) | 3 (27.3) | 0.857 (0.117-6.264) | 0.879 | 0.810 (0.108-6.063) | 0.837 |
| **Surgery** |  |  |  | 0.480 |  |  |
| Surgery | 82 (89.1) | 9 (81.8) | 1 (Ref) |  |  |  |
| biopsy | 10 (10.9) | 2 (18.2) | 1.822 (0.344-9.650) |  |  |  |
| **Chemotherapy** |  |  |  | 0.678 |  |  |
| Yes  No | 20 (21.7)  72 (78.3) | 3 (27.3)  8 (72.7) | 1.350 (0.328-5.565)  1 (Ref) |  |  |  |
| Note: PanNETs: pancreatic neuroendocrine tumors; OR: odds ratio; CI: confidence interval; NAT: neoadjuvant treatment; WHO: World Health Organization; NA: not available; Ref: reference; **^a^**Grade at first diagnosis. | | | | | | |
